# Real-world comparison of shape-sensing robotic-assisted bronchoscopy and virtual bronchoscopic navigation for peripheral pulmonary lesions: a propensity score-matched study

**DOI:** 10.1186/s12931-026-03713-3

**Published:** 2026-05-12

**Authors:** Mengzhen You, Yalun Li, Liguo Wang, Fansen Li, Shan Xu, Li Xu, Bingbing Ren, Jisong Zhang, Enguo Chen

**Affiliations:** 1 Department of Pulmonary and Critical Care Medicine, Sir Run Run Shaw Hospital, School of Medicine, Regional Medical Center for National Institute of Respiratory Disease, Zhejiang University, No.3 Qingchun East Road, Hangzhou, 310016 People’s Republic of China; 2 School of Medicine, Zhejiang University, Hangzhou, People’s Republic of China; 3 Department of Cardiology, Sir Run Run Shaw Hospital, Zhejiang University School of Medicine, Hangzhou, China; 4 Zhejiang Key Laboratory of Cardiovascular Intervention and Precision Medicine, Hangzhou, China; 5 Engineering Research Center for Cardiovascular Innovative Devices of Zhejiang Province, Hangzhou, China; 6 Central Lab of Biomedical Research Center, Sir Run Run Shaw Hospital, School of Medicine, Zhejiang University, Hangzhou, 310020 China

**Keywords:** Robotic-assisted bronchoscopy, Virtual bronchoscopic navigation, Peripheral pulmonary lesions, Diagnostic yield, Propensity score matching

## Abstract

**Background:**

Robotic-assisted bronchoscopy (RAB) may improve diagnosis of peripheral pulmonary lesions (PPLs), particularly when lesion localization and sampling are technically demanding. However, direct real-world comparisons between shape-sensing RAB (ss-RAB) and virtual bronchoscopic navigation (VBN) remain limited.

**Methods:**

We retrospectively reviewed patients who underwent navigational bronchoscopy for PPLs at a single center. Propensity score matching (PSM) was used to balance baseline characteristics between the ss-RAB and VBN groups. Diagnostic yield, tool-in-lesion (TIL) rate, sensitivity for malignancy, adverse events, and learning curves of ss-RAB were evaluated. Learning curves were assessed using cumulative sum analysis based on procedure time and diagnostic success.

**Results:**

Following 1:1 PSM, 234 patients were included, with 117 in each group. Final malignancy prevalence was 74.8% overall. Diagnostic yield was higher with ss-RAB than with VBN (85.5% vs. 70.1%; OR 2.39, 95% CI 1.25–4.56). TIL rate was also more frequently achieved with ss-RAB (99.1% vs. 88.9%). Sensitivity for malignancy was 95.6% for ss-RAB compared with 81.2% for VBN. The diagnostic advantage of ss-RAB was most evident in small (< 15 mm), peripheral, bronchus-sign-negative, and upper-lobe lesions, whereas performance was similar in larger or more central nodules. Procedure-related complications were infrequent and comparable between groups. Learning-curve analysis for ss-RAB showed a reduction in procedure time after approximately 23 cases and an earlier improvement in diagnostic success around the 13th case.

**Conclusions:**

In a real-world setting, ss-RAB was associated with higher diagnostic yield and TIL rates compared with the VBN-based comparator arm without compromising safety. The observed benefit of ss-RAB was greater in lesion subgroups with higher procedural difficulty. In experienced centers, procedural performance with ss-RAB improved after a relatively short learning phase.

**Supplementary Information:**

The online version contains supplementary material available at 10.1186/s12931-026-03713-3.

## Introduction

The diagnostic approach to pulmonary nodules has changed markedly over the past decade. For peripheral pulmonary lesions (PPLs), particularly those lacking a bronchus sign or adjacent to the pleura, percutaneous transthoracic needle aspiration is commonly used. However, this approach carries a meaningful risk of pneumothorax and bleeding. By contrast, conventional bronchoscopy may have lower diagnostic accuracy for PPLs, particularly when route selection through distal airways, lesion localization, and tissue sampling are challenging and more dependent on operator experience. These limitations have driven the development of navigational bronchoscopy to improve pathway planning, lesion localization, and biopsy targeting in peripheral lesions.

Navigation platforms, including virtual bronchoscopic navigation (VBN) and electromagnetic navigation bronchoscopy (ENB), particularly when combined with adjunctive imaging modalities, have shown improved pathway planning and diagnostic yield for PPLs in prior studies [1–3]. Nevertheless, performance remains suboptimal in small lesions, particularly those measuring ≤ 20 mm or located in the outer third of the lung. Reported diagnostic yields vary substantially across studies and are influenced by lesion features, operator experience, and procedural stability [4–7]. Consistent with these observations, a recent meta-analysis reported an overall diagnostic yield of only 70.9% for navigational bronchoscopy [8]. More recently, robotic-assisted bronchoscopy (RAB) has been introduced to improve distal control and procedural stability during biopsy.

Currently, three RAB systems have been cleared by the U.S. Food and Drug Administration (FDA): the Monarch system (Auris Health), the Ion system (Intuitive Surgical), and the Galaxy system (Noah Medical). Among available robotic bronchoscopy platforms, the Ion system incorporates shape-sensing technology for catheter localization and is therefore referred to as shape-sensing robotic-assisted bronchoscopy (ss-RAB). Although ss-RAB has been increasingly studied in comparison with conventional navigation platforms, evidence from Asian populations remains limited [9, 10]. In routine practice, navigation technology adoption is influenced by cost, infrastructure requirements, and ease of deployment, and VBN therefore remains widely used. Most comparative studies have focused on ss-RAB versus ENB, while direct comparisons between ss-RAB and VBN are scarce [10, 11], and their relative performance remains insufficiently defined. Therefore, we compared the clinical performance of ss-RAB and VBN in a Chinese population to provide real-world evidence that may inform selection of navigation strategies for PPLs.

## Method

### Study design

This single-center, retrospective study was conducted at the Respiratory Endoscopy Center of Sir Run Run Shaw Hospital. Consecutive adult patients (≥ 18 years) who underwent navigational bronchoscopy for PPLs between June 2024 and June 2025 were screened. Following standard pre-biopsy evaluation (Appendix E1), selection of navigation modality was individualized in routine clinical practice by the treating bronchoscopist, and no predefined allocation protocol was applied. Eligible lesions were ≤ 30 mm in maximum diameter on computed tomography (CT) and located within the lung parenchyma without endobronchial visibility proximal to the segmental bronchi. Lesions were investigated when malignancy was suspected or when a poor response to empirical anti-infective therapy was observed, defined as persistence, enlargement, or minimal interval change (< 30% reduction in maximum diameter) on follow-up CT performed 4–6 weeks after at least 10 days of treatment. Sampling of more than one target lesion during a single session was excluded because procedure time could not be reliably assigned at the lesion level; cases without biopsy and cases lacking sufficient follow-up to establish a reference diagnosis were also excluded. In addition, inclusion of more than one lesion from the same patient would introduce within-patient correlation into lesion-level propensity score matching (PSM). The study was conducted in accordance with the Declaration of Helsinki and was approved by the Institutional Review Board (Approval No. 2025-Research-1086). Written informed consent was waived because of the retrospective study design. This study is reported in accordance with the Standards for Reporting Diagnostic Accuracy Studies (STARD) guidelines.

### Procedure details

#### Anesthesia and preprocedural planning

Anesthetic management, including general anesthesia with neuromuscular blockade, endotracheal intubation and mechanical ventilation, was provided by the anesthesiology team. Volume-controlled ventilation was used with a tidal volume of 8–10 mL/kg and positive end-expiratory pressure maintained at 8–15 cm H₂O to minimize atelectasis. Anesthetic care, airway management, and ventilatory support were applied consistently throughout the study period. All navigational procedures were performed by two interventional bronchoscopist, both of whom had substantial prior experience with VBN but no prior experience with ss-RAB at the start of the study. Thin-slice chest CT data were used for preprocedural planning in all cases, and were reconstructed to generate navigation pathways to the target lesion.

#### Navigation systems

Navigational bronchoscopy was performed using either a RAB system or a VBN platform. RAB was performed using the Ion™ system (Intuitive Surgical, Sunnyvale, CA, USA), which is equipped with a 3.5-mm articulating catheter. Preprocedural planning was performed using the PlanPoint™ platform (Intuitive Surgical).

VBN was performed using the LungPro™ system (Broncus Technologies, Mountain View, CA, USA), which combines CT-based virtual navigation with optical tracking and augmented reality. Preprocedural imaging data were processed using the LungPoint™ platform (Broncus Technologies).

#### Lesion localization and tissue sampling

After navigation along the planned pathway, lesion localization and tissue sampling followed a standardized workflow in both groups. During navigation, the RAB system utilized a robotic catheter with an integrated vision probe, whereas the VBN system relied on a flexible bronchoscope with a 2.0-mm working channel (BF-P290; Olympus, Tokyo, Japan). R-EBUS (UM-S20-17 S; Olympus) was performed in all cases to assess the relationship between the airway and the target lesion. Fixed CBCT (Siemens Healthineers, Forchheim, Germany) was used as a non-integrated intraprocedural imaging modality to assess lesion localization and confirm tool-in-lesion (TIL) positioning before biopsy. Rapid on-site evaluation (ROSE) was routinely performed in all procedures in both groups to assess specimen adequacy as part of the standard workflow at our center.

Transbronchial biopsy was performed using one or more sampling tools, including Flexision™ needles (19G, 21G, 23G; Ion™, Intuitive Surgical) for ss-RAB and FlexNeedle™ (18G; LungPro™, Broncus Technologies) for VBN, respectively; conventional biopsy forceps (1.9 mm; Porco), fine biopsy forceps (1.5 mm; Olympus), and fine cryobiopsy probes (1.1 mm; Erbe). Additional sampling methods included cytology brushing (JRS-A, 1.8 × 1200 mm; Jingrui Medical Technology) and bronchial lavage. The choice of biopsy tools was made at the bronchoscopist’s discretion.

### Study outcomes

The primary outcome was diagnostic yield, defined as the proportion of biopsied lesions with a definitive diagnosis among all target lesions undergoing biopsy. A strict definition based on American Thoracic Society/American College of Chest Physicians (ATS/ACCP) statement was applied [12]. Biopsy specimens were considered diagnostic if they demonstrated malignant pathology or a specific benign diagnosis, including benign lung tumors (e.g., hamartoma), organizing pneumonia, granulomatous disease, or infectious etiologies (including fungal, mycobacterial, or bacterial infections). All other findings, including nonspecific inflammation, atypia not diagnostic of malignancy, or normal lung parenchyma, were classified as nondiagnostic.

Secondary outcomes included diagnostic sensitivity for malignancy, TIL confirmation, procedure-related adverse events, and learning curves of ss-RAB based on procedure time and diagnostic yield. Final diagnosis was established using clinical, pathological, and radiologic follow-up of at least 6 months. Non-diagnostic biopsy results later confirmed as malignant during follow-up were included in the denominator for sensitivity for malignancy [12]. TIL was defined as three-dimensional confirmation of the biopsy tool tip located within the target lesion on intraprocedural CBCT, assessed on multiplanar reconstructions in the axial, coronal, and sagittal planes (Figure E1). Adverse events occurring within 30 days after the procedure were assessed and graded according to the Common Terminology Criteria for Adverse Events (CTCAE) [13]. Procedure time was evaluated in the ss-RAB group only and defined as the interval from catheter in to catheter out.

### Statistical analysis

Continuous variables are presented as median with interquartile range (IQR), and categorical variables are summarized as counts and percentages. Comparisons between groups were performed using the Wilcoxon rank-sum test for continuous variables and the chi-square test or Fisher’s exact test for categorical variables.

PSM was performed to reduce confounding related to the nonrandomized study design. Propensity scores were estimated using a multivariable logistic regression model incorporating age, sex, and lesion characteristics (size, density, lobar location, outer-third location, presence of a bronchus sign, and distance from the pleura), selected a priori based on prior studies [14–16]. Patients were matched 1:1 using nearest-neighbor matching with a caliper of 0.2 of the standard deviation of the logit of the propensity score. After matching, binary outcomes were performed using conditional logistic regression, accounting for the matched design. In prespecified subgroup analyses, Firth’s penalized logistic regression was used to estimate odds ratio (OR) and corresponding confidence intervals to account for small sample sizes and potential separation.

Learning curves of ss-RAB were assessed using cumulative sum (CUSUM) analysis. Cases were analyzed in chronological order using the original dataset prior to PSM. For procedure time, CUSUM values represented the cumulative deviation from the mean procedure time. For diagnostic yield, a target-based CUSUM approach was applied. The target diagnostic yield (p_0_) was set at 80% based on previously reported diagnostic yields for PPLs [17, 18]. For CUSUM analysis, malignant or specific benign pathology was considered a success and non-diagnostic results a failure. The nadir of the curve was interpreted as the point at which diagnostic performance reached the predefined target.

## Result

### Baseline characteristics

**Fig. 1:**
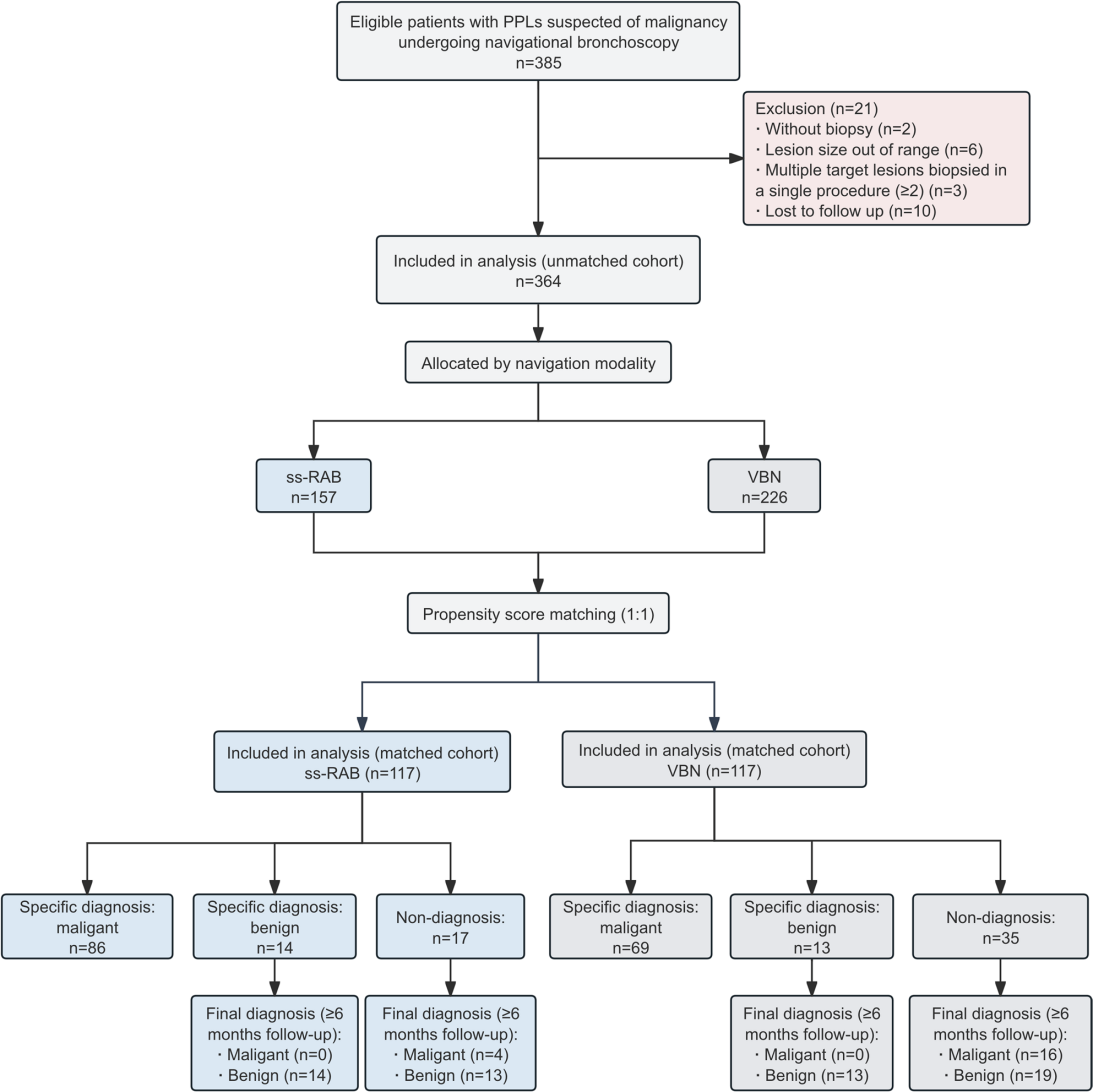
Flow diagram of patient inclusion and diagnostic classification according to biopsy results and follow-up. Ss-RAB = shape-sensing robotic-assisted bronchoscopy; VBN = virtual bronchoscopic navigation

Between June 2024 and June 2025, a total of 385 patients who underwent bronchoscopic navigation with biopsy using either the ss-RAB or the VBN at our center were assessed for eligibility. After exclusion of 21 ineligible cases due to absence of biopsy, lesions outside the predefined size range, biopsy of multiple target lesions in a single procedure, or insufficient follow-up, 364 patients were included in the unmatched cohort (Fig. 1). Baseline demographic and lesion characteristics of patients who underwent biopsy are summarized in Table 1. Before PSM, lesion characteristics differed between groups. Lesions in the VBN group were larger in size compared with those in the ss-RAB group (18.1 mm vs. 12.4 mm, *P* < .001), and a bronchus sign was more common in the VBN group (79.9% vs. 64.0%, *P* = .001). After 1:1 PSM, 117 patients remained in each group. Sampling strategies in the matched cohort are summarized in Table E1. Following matching, baseline demographic and lesion characteristics were well balanced, with no statistically significant differences observed across all covariates (Figure E2).

**Table 1:** Baseline demographic and lesion characteristics

Variable	Before Matching				After Matching			
	Overall (*n*=364)	ss-RAB (*n*=150)	VBN (*n*=214)	*P* value	Overall (*n*=234)	ss-RAB (*n*=117)	VBN (*n*=117)	*P* value
Age, years, mean (SD)	64.80 (11.37)	63.77 (11.38)	65.53 (11.33)	0.147	64.40 (11.65)	64.39 (11.75)	64.41 (11.61)	0.991
Sex, n (%)				0.582				0.895
Male	204 (56.0)	81 (54.0)	123 (57.5)		136 (58.1)	67 (57.3)	69 (59.0)	
Female	160 (44.0)	69 (46.0)	91 (42.5)		98 (41.9)	50 (42.7)	48 (41.0)	
Body mass index, kg/m2, n (%)				0.539				0.615
<24	238 (65.4)	103 (68.7)	135 (63.1)		151 (64.5)	79 (67.5)	72 (61.5)	
<28	101 (27.7)	38 (25.3)	63 (29.4)		69 (29.5)	32 (27.4)	37 (31.6)	
≥28	25 (6.9)	9 (6.0)	16 (7.5)		14 (6.0)	6 (5.1)	8 (6.8)	
Smoking status, n (%)				0.112				0.188
Never	210 (57.7)	96 (64.0)	114 (53.3)		130 (55.6)	72 (61.5)	58 (49.6)	
Previous	98 (26.9)	33 (22.0)	65 (30.4)		66 (28.2)	27 (23.1)	39 (33.3)	
Current	56 (15.4)	21 (14.0)	35 (16.4)		38 (16.2)	18 (15.4)	20 (17.1)	
Prior lung malignancy, n (%)				0.072				0.700
Yes	48 (13.2)	26 (17.3)	22 (10.3)		31 (13.2)	17 (14.5)	14 (12.0)	
No	316 (86.8)	124 (82.7)	192 (89.7)		203 (86.8)	100 (85.5)	103 (88.0)	
Lesion size (largest diameter), mm, median [IQR]	15.21 [11.41, 20.19]	12.40 [9.42, 15.69]	18.11 [13.26, 24.52]	< 0.001	13.64 [11.12, 16.55]	13.31 [11.00, 16.64]	13.79 [11.24, 16.30]	0.516
Lesion location (outer 1/3 of the chest), n (%)				0.373				0.781
Yes	249 (68.4)	107 (71.3)	142 (66.4)		157 (67.1)	80 (68.4)	77 (65.8)	
No	115 (31.6)	43 (28.7)	72 (33.6)		77 (32.9)	37 (31.6)	40 (34.2)	
Lesion density, n (%)				0.201				0.998
Cavitary	22 (6.0)	5 (3.3)	17 (7.9)		10 (4.3)	5 (4.3)	5 (4.3)	
Solid	247 (67.9)	101 (67.3)	146 (68.2)		163 (69.7)	81 (69.2)	82 (70.1)	
Part-solid	49 (13.5)	21 (14.0)	28 (13.1)		31 (13.2)	16 (13.7)	15 (12.8)	
Pure ground-glass	46 (12.6)	23 (15.3)	23 (10.7)		30 (12.8)	15 (12.8)	15 (12.8)	
Lesion specific lobes, n (%)				0.903				0.896
Right upper	126 (34.6)	52 (34.7)	74 (34.6)		77 (32.9)	42 (35.9)	35 (29.9)	
Right middle	19 (5.2)	7 (4.7)	12 (5.6)		13 (5.6)	6 (5.1)	7 (6.0)	
Right lower	67 (18.4)	31 (20.7)	36 (16.8)		46 (19.7)	23 (19.7)	23 (19.7)	
Left upper	91 (25.0)	36 (24.0)	55 (25.7)		55 (23.5)	26 (22.2)	29 (24.8)	
Left lower	61 (16.8)	24 (16.0)	37 (17.3)		43 (18.4)	20 (17.1)	23 (19.7)	
Bronchus sign, n (%)				0.001				0.671
Yes	267 (73.4)	96 (64.0)	171 (79.9)		162 (69.2)	79 (67.5)	83 (70.9)	
No	97 (26.6)	54 (36.0)	43 (20.1)		72 (30.8)	38 (32.5)	34 (29.1)	
Lesion distance from the pleura, mm, median [IQR]	11.58 [3.64, 20.24]	10.10 [3.00, 18.44]	12.46 [3.86, 21.11]	0.191	11.94 [4.13, 20.96]	10.93 [3.93, 19.45]	12.62 [5.22, 21.66]	0.394

### Diagnostic performance

**Fig. 2:**
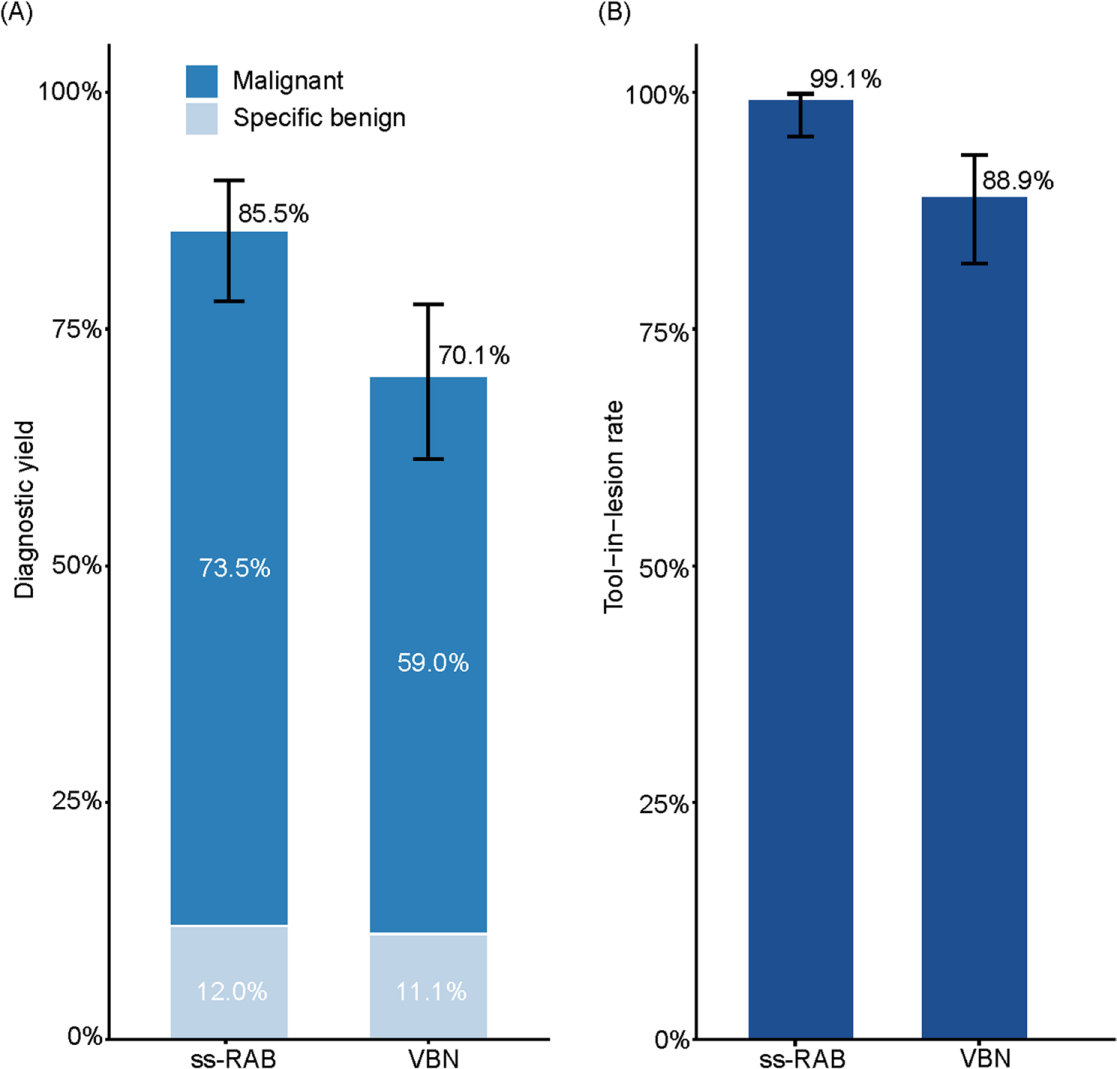
Diagnostic yield and tool-in-lesion rate by navigation modality. **A** Overall diagnostic yield stratified by navigation modality, with stacked bars showing the proportions of malignant and specific benign diagnoses. Error bars indicate 95% confidence intervals for overall diagnostic yield. **B** Tool-in-lesion rate by navigation modality, with error bars indicating 95% confidence intervals. Ss-RAB = shape-sensing robotic-assisted bronchoscopy; VBN = virtual bronchoscopic navigation

In the matched cohort, final malignancy prevalence was 74.8% overall, including 76.9% in the ss-RAB group and 72.6% in the VBN group (Table 2). Overall diagnostic yield was higher with ss-RAB than with VBN (85.5% vs. 70.1%), corresponding to an odds ratio of 2.39 (95% CI, 1.25–4.56) (Fig. 2A). A higher proportion of malignant diagnoses was observed in the ss-RAB group (73.5% vs. 59.0%), whereas the proportion of specific benign diagnoses was similar between groups (12.0% vs. 11.1%).

**Table 2:** Summary of biopsy pathology, final diagnosis of non-diagnostic cases, and diagnostic performance in the matched cohort

Biopsy pathology	ss-RAB (*n* = 117)	VBN (*n* = 117)	*P* value
Malignant			
Adenocarcinoma	74	46	
Squamous cell carcinoma	7	15	
Metastatic cancer	2	5	
Small cell carcinoma	2	3	
Lymphoma	1	0	
Specific benign			
Granulomatous disease	7	7	
Fungal	4	2	
Organising pneumonia	2	3	
Amyloidosis	1	1	
Non-diagnostic			
Atypical cells	0	3	
Normal respiratory tissue	9	17	
Nonspecific inflammation	8	15	
Final diagnosis of non-diagnostic cases at ≥ 6 months			
Malignant	4	16	
Benign	13	19	
Diagnostic performance			
Strict diagnostic yield	85.5%	70.1%	0.005
Sensitivity for malignancy	95.6%	81.2%	0.004
Cancer prevalence	76.9%	72.6%	0.452

Data are presented as number of lesions unless otherwise specified

Strict diagnostic yield was defined as the proportion of lesions with malignant pathology or a specific benign diagnosis at biopsy. Sensitivity for malignancy = biopsy malignant / (biopsy malignant + non-diagnostic lesions later confirmed as malignant). *Ss-RAB* shape-sensing robotic-assisted bronchoscopy, *VBN* virtual bronchoscopic navigation

**Fig. 3:**
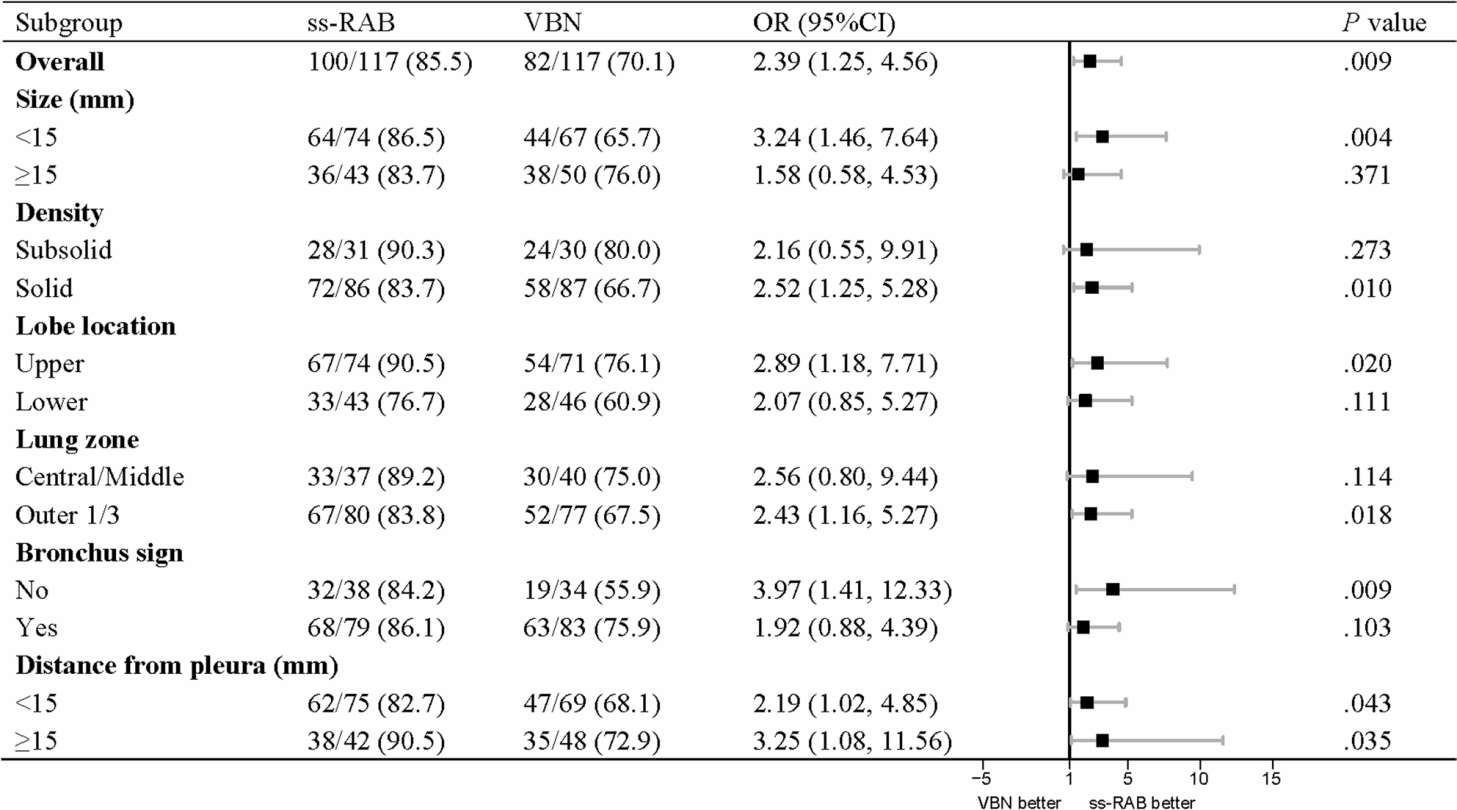
Subgroup analysis of diagnostic yield comparing ss-RAB and VBN. OR and 95% confidence intervals are shown for prespecified subgroups, with VBN as the reference category. Subsolid lesions include part-solid and pure ground-glass lesions. Solid lesions include solid and cavitary lesions. OR = odds ratio; ss-RAB = shape-sensing robotic-assisted bronchoscopy; VBN = virtual bronchoscopic navigation

Consistent with the observed difference in diagnostic yield, TIL rate was achieved more frequently with ss-RAB than with VBN (99.1% vs. 88.9%) (Fig. 2B). Using the final clinical diagnosis established after at least 6 months of follow-up as the reference standard, sensitivity for malignancy was 95.6% in the ss-RAB group and 81.2% in the VBN group (Table 2). Subgroup analyses further showed that the diagnostic advantage of ss-RAB was greatest in small (< 15 mm), peripheral, bronchus-sign-negative and upper-lobe lesions. In contrast, no statistically significant incremental benefit of ss-RAB was observed in larger lesions (≥ 15 mm), or lesions located in the central or middle lung zones (Fig. 3).

### Safety

Procedure-related complications within 30 days were uncommon in both groups and occurred at similar rates between ss-RAB and VBN. Pneumothorax was observed in one patient in the VBN group and in none of the ss-RAB cases. Bleeding events were infrequent and were managed endoscopically without the need for additional interventions. No severe bleeding events or procedure-related mortality were observed (Table E2).

### Learning curve analysis

**Fig. 4:**
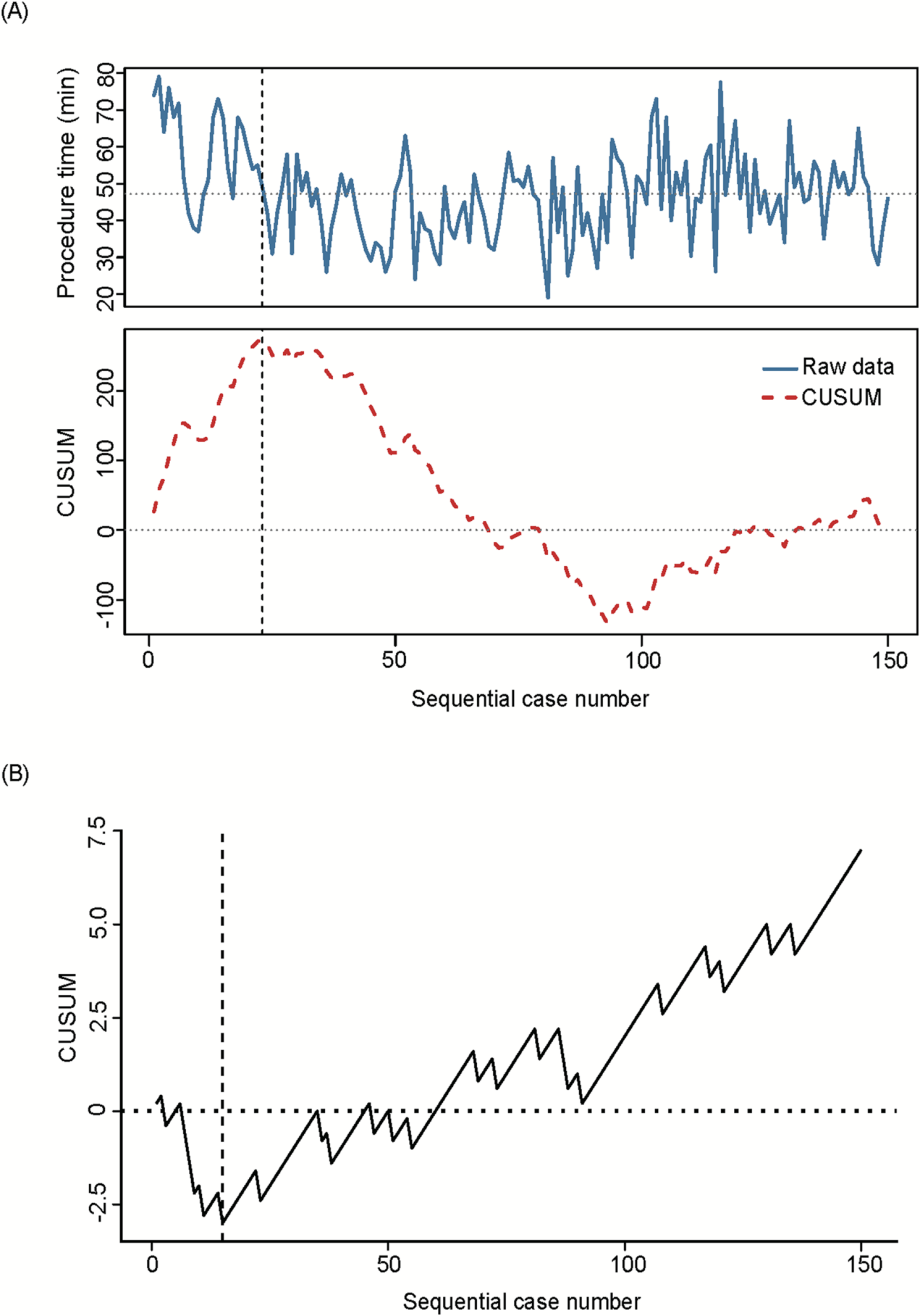
Learning curves of ss-RAB based on procedure time and diagnostic yield. **A** Procedure time. **B** Diagnostic yield. Ss-RAB = shape-sensing robotic-assisted bronchoscopy

CUSUM analysis based on procedure time demonstrated a clear temporal change in procedural performance across sequential cases (Fig. 4A). Procedure time was longer during the early phase and gradually decreased as case numbers increased. A change point was observed at approximately the 23rd case, after which procedure duration showed reduced variability and generally remained shorter than during the initial learning phase. CUSUM analysis based on diagnostic success is shown in Fig. 4B. An initial downward deflection was observed during the early cases, followed by a gradual upward trend. A change in curve direction was observed around the 13th case, suggesting improvement in diagnostic performance over time.

## Discussion

In this real-world, propensity score-matched study, ss-RAB was associated with a higher diagnostic yield and TIL rate than the VBN-based comparator arm for PPLs, with similar safety. Notably, the diagnostic advantage of ss-RAB was most pronounced in small, peripheral, upper-lobe, and bronchus-sign-negative lesions. In addition, CUSUM analysis demonstrated a clear temporal change in procedural performance during early experience with ss-RAB.

To our knowledge, this is the first large real-world study to directly compare ss-RAB with a VBN-based bronchoscopy workflow. In a recent meta-analysis, the pooled diagnostic yield of ss-RAB for PPLs was approximately 80.1% [19], and the diagnostic yield observed in the present study fell within the range of 79.3% to 91.4% reported in prior single-arm ss-RAB studies [20–23]. In contrast, reported diagnostic yields for VBN-based approaches have varied widely, ranging from 55.3% to 84.6% across published studies [24–27]. Across studies, differences in diagnostic yield definitions, use of adjunctive imaging modalities, sampling tools, and study populations make direct comparison between navigation modality difficult. The relatively high diagnostic yield observed in the non-robotic arm of our study should be interpreted in the context of a VBN-based bronchoscopy workflow supported by adjunctive CBCT and r-EBUS. By evaluating ss-RAB and a VBN-based bronchoscopy workflow within the same institution, using strict diagnostic definitions aligned with ATS/ACCP recommendations, our study provides a more standardized real-world comparison of their relative diagnostic performance.

In routine practice, both robotic and non-robotic navigational bronchoscopy approaches are available, and their comparative advantages may differ according to lesion characteristics. In our cohort, the incremental diagnostic benefit of ss-RAB was not uniform across all lesions. In larger, lower-lobe, centrally located, or bronchus-sign–positive lesions, diagnostic performance between ss-RAB and the VBN-based comparator arm was similar. In contrast, ss-RAB showed clearer benefit in small, upper-lobe, peripheral, and bronchus-sign-negative lesions. These findings suggest that the potential advantages of robotic bronchoscopy may be more relevant in lesion subgroups that require greater precision in pathway selection, catheter positioning, and tissue sampling. This may be particularly important for smaller nodules, in which even minor positional deviations can lead to sampling failure. In such settings, technical characteristics of the robotic platform, including improved flexibility and maneuverability, enhanced distal stability, shape-sensing-based catheter localization, and more consistent tool positioning during sampling, may help improve procedural performance. From a practical perspective, the higher infrastructure requirements and procedural costs of robotic bronchoscopy remain important considerations, particularly in resource-limited settings. Overall, these findings support a lesion-adapted approach to navigation strategy selection in real-world practice, although conclusions regarding the role of non-robotic approaches should remain cautious given the retrospective design and potential selection bias.

Consistent with prior observations, the diagnostic yield of ss-RAB in the present study remained lower than the TIL rate [23, 28]. This indicates that even with intraprocedural CBCT confirmation of TIL positioning, successful lesion localization does not always translate into a definitive pathological diagnosis. This should also be interpreted in the context of the strict diagnostic-yield definition used in the present study. In addition, 20 lesions with initially non-diagnostic biopsy results were subsequently confirmed as malignant during follow-up in our cohort, which may in part reflect the non-direct-visual nature of bronchoscopic biopsy, limitations of sampling tools, and intralesional heterogeneity. Prior studies have suggested that cryobiopsy may improve tissue quantity and histologic quality, possibly because it allows acquisition of larger and more representative samples [29–32]. Future improvements may therefore depend not only on navigation accuracy, but also on optimization of tissue acquisition, including more effective sampling tools, systematic multi-site sampling, and combined use of complementary biopsy modalities.

Learning curve analysis showed progressive improvement over sequential cases. Procedure time decreased after approximately the 23rd case, while the CUSUM curve for diagnostic yield changed direction earlier, around the 13th case, suggesting improvement in procedural performance over time rather than a strict stabilization threshold. Guarize et al. reported stabilization after 40–45 single-nodule procedures [33]. The earlier plateau observed in our study may reflect differences in baseline operator experience with advanced navigational bronchoscopy and familiarity with VBN prior to adoption of ss-RAB. This finding should therefore be interpreted with caution. Collectively, these findings suggest that ss-RAB can be integrated into clinical practice with a relatively short learning phase, particularly in centers with existing expertise in bronchoscopic navigation.

This study has several limitations. First, its retrospective, single high-volume center design may limit generalizability. Although PSM was used to reduce baseline imbalance, residual confounding and selection bias cannot be fully excluded, particularly given non-random modality selection and differences in sampling strategies. Second, diagnostic performance was evaluated using strict diagnostic yield rather than diagnostic accuracy, as reliable assessment of diagnostic accuracy would require more prolonged follow-up to establish the true final status of lesions with initially non-diagnostic or nonspecific biopsy findings. Finally, the learning curve analysis were derived from single-target procedures performed by experienced operators and may therefore be most applicable to centers with established expertise in advanced interventional bronchoscopy. Despite these limitations, our findings provide practical real-world evidence to inform selection of navigation strategies for PPLs. Further randomized studies with standardized follow-up are needed to better define the comparative diagnostic performance of different navigation approaches.

## Conclusion

In this real-world study, ss-RAB was associated with higher diagnostic yield and TIL rates than a VBN-based bronchoscopy workflow, with comparable safety. Its benefit was greatest in in small, peripheral, upper-lobe, and bronchus-sign-negative lesions, whereas differences were less apparent in larger or more central nodules. These results support selective application of ss-RAB and suggest that adoption may be facilitated in experienced bronchoscopy centers.

## Supplementary Information

Supplementary Material 1.

## Data Availability

Research data are stored in an institutional repository and will be shared upon request to the corresponding author.

## Abbreviations

RAB: Robotic-assisted bronchoscopy
PPLs: Peripheral pulmonary lesions
FDA: Food and Drug Administration
ss-RAB: Shape-sensing robotic-assisted bronchoscopy
VBN: Virtual bronchoscopic navigation
PSM: Propensity score matching
TIL: Tool-in-lesion
CUSUM: Cumulative sum
ENB: Electromagnetic navigation bronchoscopy
STARD: Standards for Reporting Diagnostic Accuracy Studies
CT: Computed tomography
r-EBUS: Radial endobronchial ultrasound
CBCT: Cone-beam computed tomography
ROSE: Rapid on-site evaluation
ATS: American Thoracic Society
ACCP: American College of Chest Physicians
CTCAE: Common Terminology Criteria for Adverse Events
IQR: Interquartile range

## References

[1] Zheng X, Cao L, Zhang Y, Xie F, Yang H, Liu J, et al. A Novel Electromagnetic Navigation Bronchoscopy System for the Diagnosis of Peripheral Pulmonary Nodules: A Randomized Clinical Trial. Ann Am Thorac Soc. 2022;19(10):1730–9. 35679184 10.1513/AnnalsATS.202109-1071OC

[2] Matsumoto Y, Izumo T, Sasada S, Tsuchida T, Ohe Y. Diagnostic utility of endobronchial ultrasound with a guide sheath under the computed tomography workstation (ziostation) for small peripheral pulmonary lesions. Clin Respir J. 2017;11(2):185–92. 26072931 10.1111/crj.12321

[3] Giri M, Dai H, Puri A, Liao J, Guo S. Advancements in navigational bronchoscopy for peripheral pulmonary lesions: A review with special focus on virtual bronchoscopic navigation. Front Med (Lausanne). 2022;9:989184. 36300190 10.3389/fmed.2022.989184 PMC9588954

[4] Khandhar SJ, Bowling MR, Flandes J, Gildea TR, Hood KL, Krimsky WS, et al. Electromagnetic navigation bronchoscopy to access lung lesions in 1,000 subjects: first results of the prospective, multicenter NAVIGATE study. BMC Pulm Med. 2017;17(1):59. 28399830 10.1186/s12890-017-0403-9 PMC5387322

[5] Patrucco F, Daverio M, Airoldi C, Falaschi Z, Longo V, Gavelli F, et al. 4D Electromagnetic Navigation Bronchoscopy for the Sampling of Pulmonary Lesions: First European Real-Life Experience. Lung. 2021;199(5):493–500. 34562105 10.1007/s00408-021-00477-z PMC8510943

[6] Sun J, Criner GJ, Dibardino D, Li S, Nader D, Lam B, et al. Efficacy and safety of virtual bronchoscopic navigation with fused fluoroscopy and vessel mapping for access of pulmonary lesions. Respirology. 2022;27(5):357–65. 35212090 10.1111/resp.14224

[7] Kim YW, Kim HJ, Kwon BS, Lee YJ, Song MJ, Yoon SH, et al. Diagnostic Yield and Synergistic Impact of Needle Aspiration and Forceps Biopsy With Electromagnetic Navigation Bronchoscopy for Peripheral Pulmonary Lesions: A Randomized Controlled Trial. Chest. 2025;168(1):236–47. 39993594 10.1016/j.chest.2025.02.015

[8] Kops SEP, Heus P, Korevaar DA, Damen JAA, Idema DL, Verhoeven RLJ, et al. Diagnostic yield and safety of navigation bronchoscopy: A systematic review and meta-analysis. Lung Cancer. 2023;180:107196. 37130440 10.1016/j.lungcan.2023.107196

[9] LowSW, Abdeljaleel F, Kemper B, Wang Y, Wang X, Yurosko C et al. Shape-Sensing Robotic-Assisted Bronchoscopy vs. Electromagnetic Robotic-Assisted Bronchoscopy-A Comparative Cohort Study. J Clin Med. 2026;15(3). 10.3390/jcm15031284. 10.3390/jcm15031284 PMC12898066 41682967

[10] Paez R, Lentz RJ, Duke JD, Siemann JK, Salmon C, Dahlberg GJ, et al. Robotic versus Electromagnetic Bronchoscopy for Peripheral Pulmonary Lesions: A Randomized Trial (RELIANT). Am J Respir Crit Care Med. 2025;211(9):1644–51. 40460390 10.1164/rccm.202409-1846OC PMC12432399

[11] Low SW, Lentz RJ, Chen H, Katsis J, Aboudara MC, Whatley S, et al. Shape-Sensing Robotic-Assisted Bronchoscopy vs Digital Tomosynthesis-Corrected Electromagnetic Navigation Bronchoscopy: A Comparative Cohort Study of Diagnostic Performance. Chest. 2023;163(4):977–84. 36441041 10.1016/j.chest.2022.10.019

[12] Gonzalez AV, Silvestri GA, Korevaar DA, Gesthalter YB, Almeida ND, Chen A, et al. Assessment of Advanced Diagnostic Bronchoscopy Outcomes for Peripheral Lung Lesions: A Delphi Consensus Definition of Diagnostic Yield and Recommendations for Patient-centered Study Designs. An Official American Thoracic Society/American College of Chest Physicians Research Statement. Am J Respir Crit Care Med. 2024;209(6):634–46. 38394646 10.1164/rccm.202401-0192ST PMC10945060

[13] Freites-Martinez A, Santana N, Arias-Santiago S, Viera A. Using the Common Terminology Criteria for Adverse Events (CTCAE - Version 5.0) to Evaluate the Severity of Adverse Events of Anticancer Therapies. Actas Dermosifiliogr (Engl Ed). 2021;112(1):90–2. 32891586 10.1016/j.ad.2019.05.009

[14] Ali MS, Trick W, Mba BI, Mohananey D, Sethi J, Musani AI. Radial endobronchial ultrasound for the diagnosis of peripheral pulmonary lesions: A systematic review and meta-analysis. Respirology. 2017;22(3):443–53. 28177181 10.1111/resp.12980

[15] Ito T, Matsumoto Y, Okachi S, Nishida K, Tanaka M, Imabayashi T, et al. A Diagnostic Predictive Model of Bronchoscopy with Radial Endobronchial Ultrasound for Peripheral Pulmonary Lesions. Respiration. 2022;101(12):1148–56. 36327951 10.1159/000526574

[16] Ost DE, Ernst A, Lei X, Kovitz KL, Benzaquen S, Diaz-Mendoza J, et al. Diagnostic Yield and Complications of Bronchoscopy for Peripheral Lung Lesions. Results of the AQuIRE Registry. Am J Respir Crit Care Med. 2016;193(1):68–77. 26367186 10.1164/rccm.201507-1332OC PMC4731617

[17] Fernandez-Bussy S, Yu Lee-Mateus A, Barrios-Ruiz A, Valdes-Camacho S, Lin K, Ibrahim MI, et al. Diagnostic performance of shape-sensing robotic-assisted bronchoscopy for pleural-based and fissure-based pulmonary lesions. Thorax. 2025;80(3):150–8. 39837619 10.1136/thorax-2024-222502

[18] Ali MS, Ghori UK, Wayne MT, Shostak E, De Cardenas J. Diagnostic Performance and Safety Profile of Robotic-assisted Bronchoscopy: A Systematic Review and Meta-Analysis. Ann Am Thorac Soc. 2023;20(12):1801–12. 37769170 10.1513/AnnalsATS.202301-075OC

[19] Li X, Bai J, Zhou X, Wang T, Zhang Y, Hu Y. Diagnostic performance and safety for robotic-assisted bronchoscopy in pulmonary nodules: a systematic review and meta-analysis. Int J Surg. 2025;111(6):4020–32. 40358662 10.1097/JS9.0000000000002423 PMC12165526

[20] Fielding DIK, Bashirzadeh F, Son JH, Todman M, Chin A, Tan L, et al. First Human Use of a New Robotic-Assisted Fiber Optic Sensing Navigation System for Small Peripheral Pulmonary Nodules. Respiration. 2019;98(2):142–50. 31352444 10.1159/000498951

[21] Styrvoky K, Schwalk A, Pham D, Chiu HT, Rudkovskaia A, Madsen K, et al. Shape-Sensing Robotic-Assisted Bronchoscopy with Concurrent use of Radial Endobronchial Ultrasound and Cone Beam Computed Tomography in the Evaluation of Pulmonary Lesions. Lung. 2022;200(6):755–61. 36369295 10.1007/s00408-022-00590-7

[22] Husta BC, Cheng GZ, Batra H, Reisenauer JS, Bartek WM, Kalchiem-Dekel O, et al. Shape-sensing robotic-assisted bronchoscopy with integrated mobile cone-beam CT for small nodules: results from the prospective multicentre CONFIRM study. Thorax. 2026;81(3):267–75. 41698810 10.1136/thorax-2025-223272

[23] ChanLT, Lau KKW, Orton CM, Temov K, Tana A, Baboolal I et al. Tool in lesion verification of shape-sensing robotic-assisted bronchoscopy with cone beam CT in sampling peripheral pulmonary nodules. Thorax. 2025. 10.1136/thorax-2025-223631. 10.1136/thorax-2025-223631 PMC13217089 41391887

[24] Zheng X, Zhong C, Xie F, Li S, Wang G, Zhang L, et al. Virtual bronchoscopic navigation and endobronchial ultrasound with a guide sheath without fluoroscopy for diagnosing peripheral pulmonary lesions with a bronchus leading to or adjacent to the lesion: A randomized non-inferiority trial. Respirology. 2023;28(4):389–98. 36356596 10.1111/resp.14405

[25] OkiM, Saka H, Imabayashi T, Himeji D, Nishii Y, Nakashima H et al. Guide sheath versus non-guide sheath method for endobronchial ultrasound-guided biopsy of peripheral pulmonary lesions: a multicentre randomised trial. Eur Respir J. 2022;59(5). 10.1183/13993003.01678-2021. 10.1183/13993003.01678-2021 34625482

[26] Kawakita N, Takehara E, Takeuchi T, Fujimoto K, Sakamoto S, Sumitomo H, et al. Advantages of a larger working channel diameter of ultrathin bronchoscope in cone-beam computed tomography-guided transbronchial biopsy for diagnosing peripheral lung lesions. Lung Cancer. 2025;202:108483. 40056873 10.1016/j.lungcan.2025.108483

[27] Asano F, Shinagawa N, Ishida T, Shindoh J, Anzai M, Tsuzuku A, et al. Virtual bronchoscopic navigation combined with ultrathin bronchoscopy. A randomized clinical trial. Am J Respir Crit Care Med. 2013;188(3):327–33. 23600452 10.1164/rccm.201211-2104OC

[28] Chen AC, Pastis NJ Jr., Mahajan AK, Khandhar SJ, Simoff MJ, Machuzak MS, et al. Robotic Bronchoscopy for Peripheral Pulmonary Lesions: A Multicenter Pilot and Feasibility Study (BENEFIT). Chest. 2021;159(2):845–52. 32822675 10.1016/j.chest.2020.08.2047 PMC7856527

[29] Chen-YostHI, Pang J, Hao W, Geng Y, Keil S, Sherman A et al. Accuracy of Rapid On-Site Evaluation in Robotic-Assisted Bronchoscopy Fine Needle Aspirations of Lung Nodules. J Bronchol Interv Pulmonol. 2026;33(2). 10.1097/LBR.0000000000001058. 10.1097/LBR.0000000000001058 41718084

[30] Husta BC, Ganjaei KG, Knezevic A, Aly RG, Fanaroff R, Lee RP, et al. A Prospective Study of Safety and the Incremental Diagnostic Value of Transbronchial Cryobiopsy Incorporated into Robotic-Assisted Bronchoscopy in a Cancer Population. Lung. 2025;204(1):1. 41413664 10.1007/s00408-025-00863-x PMC12784132

[31] Kalchiem-Dekel O, Rakocevic R, Toumbacaris N, Tan KS, Nadig TR, Adusumilli PS, et al. Robotic-assisted bronchoscopy for histopathologic subtyping of primary lung adenocarcinoma. Lung Cancer. 2025;207:108681. 40749261 10.1016/j.lungcan.2025.108681 PMC12379800

[32] Oberg CL, Lau RP, Folch EE, He T, Ronaghi R, Susanto I, et al. Novel Robotic-Assisted Cryobiopsy for Peripheral Pulmonary Lesions. Lung. 2022;200(6):737–45. 36216921 10.1007/s00408-022-00578-3 PMC9675683

[33] GuarizeJ, Bertolaccini L, Bardoni C, Donghi SM, Spaggiari L. Characterizing the Learning Curve of ION Robotic Bronchoscopy: A CUSUM-Based Analysis of Diagnostic Yield. J Bronchol Interv Pulmonol. 2026;33(1). 10.1097/LBR.0000000000001043. 10.1097/LBR.0000000000001043 41216684

