# Supplementary material for: Real-world comparison of shape-sensing robotic-assisted bronchoscopy and virtual bronchoscopic navigation for peripheral pulmonary lesions: a propensity score-matched study
Source: Respir Res. 2026 May 12;27:280. doi: 10.1186/s12931-026-03713-3 (PMC13366844; doi:10.1186/s12931-026-03713-3)
Supplement: Supplementary file 1 — Supplementary Material 1. [file 12931_2026_3713_MOESM1_ESM.pdf]

Real-World Comparison of Shape-Sensing Robotic-Assisted Bronchoscopy and Virtual Bronchoscopic Navigation for Peripheral Pulmonary Lesions: A Propensity Score-Matched Study

Mengzhen You[1,2#]; Yalun Li[1,2#]; Liguo Wang[3,4,5#]; Fansen Li[1]; Shan Xu[1]; Li Xu[1]; Bingbing Ren[1,6*]; Jisong Zhang[1*]; Enguo Chen[1*]

[#] Mengzhen You, Yalun Li and Liguo Wang contributed equally to this work.

Institutions

[1]Department of Pulmonary and Critical Care Medicine, Regional Medical Center for National Institute of Respiratory Disease, Sir Run Run Shaw Hospital, School of Medicine, Zhejiang University, Hangzhou 310016, People's Republic of China;

[2]School of Medicine, Zhejiang University, Hangzhou, People's Republic of China

[3]Department of Cardiology, Sir Run Run Shaw Hospital, Zhejiang University School of Medicine, Hangzhou, China

[4]Zhejiang Key Laboratory of Cardiovascular Intervention and Precision Medicine Hangzhou, China

[5]Engineering Research Center for Cardiovascular Innovative Devices of Zhejiang Province, Hangzhou, China

[6]Central Lab of Biomedical Research Center, Sir Run Run Shaw Hospital, School of Medicine, Zhejiang University, Hangzhou 310020, China

*Corresponding authors: Enguo Chen, Department of Pulmonary and Critical Care Medicine, Regional Medical Center for National Institute of Respiratory Disease, Sir Run Run Shaw Hospital, School of Medicine, Zhejiang University, No.3 Qingchun East Road, Hangzhou 310016, People's Republic of China (Email: cheneg@zju.edu.cn). Jisong Zhang, Department of Pulmonary and Critical Care Medicine, Regional Medical Center for National Institute of Respiratory Disease, Sir Run Run Shaw Hospital, School of Medicine, Zhejiang University, No.3 Qingchun East Road, Hangzhou 310016, People's Republic of China (Email: zhangjisong@zju.edu.cn). Bingbing Ren, Department of Pulmonary and Critical Care Medicine, Regional Medical Center for National Institute of Respiratory Disease, Sir Run Run Shaw Hospital, School of Medicine, Zhejiang University, No.3 Qingchun East Road, Hangzhou 310016, People's Republic of China (Email: renbb@zju.edu.cn).

Table E1. Sampling strategies used in the matched cohort.

Sampling strategy	ss-RAB (n=117)	VBN (n=117)	*P* value
Needle alone	6 (5.1%)	2 (1.7%)	.281
Forceps alone	40 (34.2%)	51 (43.6%)	.180
Cryobiopsy alone	46 (39.3%)	45 (38.5%)	1.000
Needle + forceps	12 (10.3%)	10 (8.5%)	.823
Needle + cryobiopsy	7 (6.0%)	7 (6.0%)	1.000
Forceps + cryobiopsy	3 (2.6%)	2 (1.7%)	1.000
Needle + forceps + cryobiopsy	3 (2.6%)	0 (0.0%)	.247

Data are presented as n (%). P values were calculated using Fisher's exact test to compare the proportion of each sampling strategy between the two groups. ss-RAB = shape-sensing robotic-assisted bronchoscopy; VBN = virtual bronchoscopic navigation.

Table E2. Procedure-related complications by navigation modality.

Complication	ss-RAB	VBN	*P* value
Pneumothorax	0/117 (0.0)	1/117 (0.9)	-
Bleeding	3/117 (2.6)	6/117 (5.1)	.330
CTCAE grade ≥3	0/117 (0.0)	1/117 (0.9)	-

Data are presented as number (%). Complications within 30 days were graded according to CTCAE version 5.0. Ss-RAB = shape-sensing robotic-assisted bronchoscopy; VBN = virtual bronchoscopic navigation.

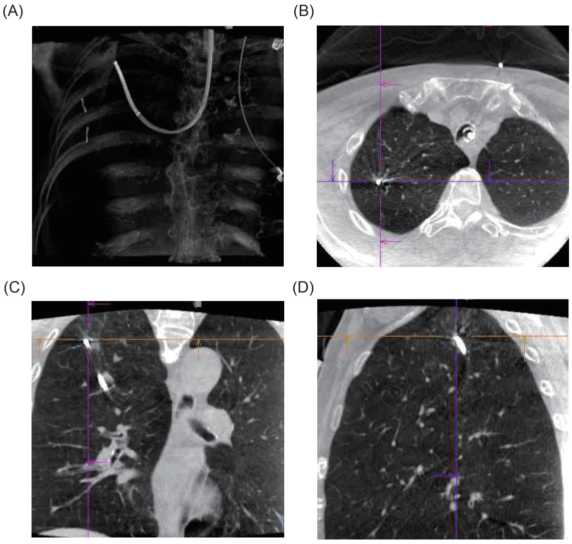

Figure E1. Intraprocedural confirmation of tool-in-lesion positioning by fluoroscopy and CBCT.

(A) Fluoroscopy view; (B) Axial view; (C) Coronal view; (D) Sagittal view.

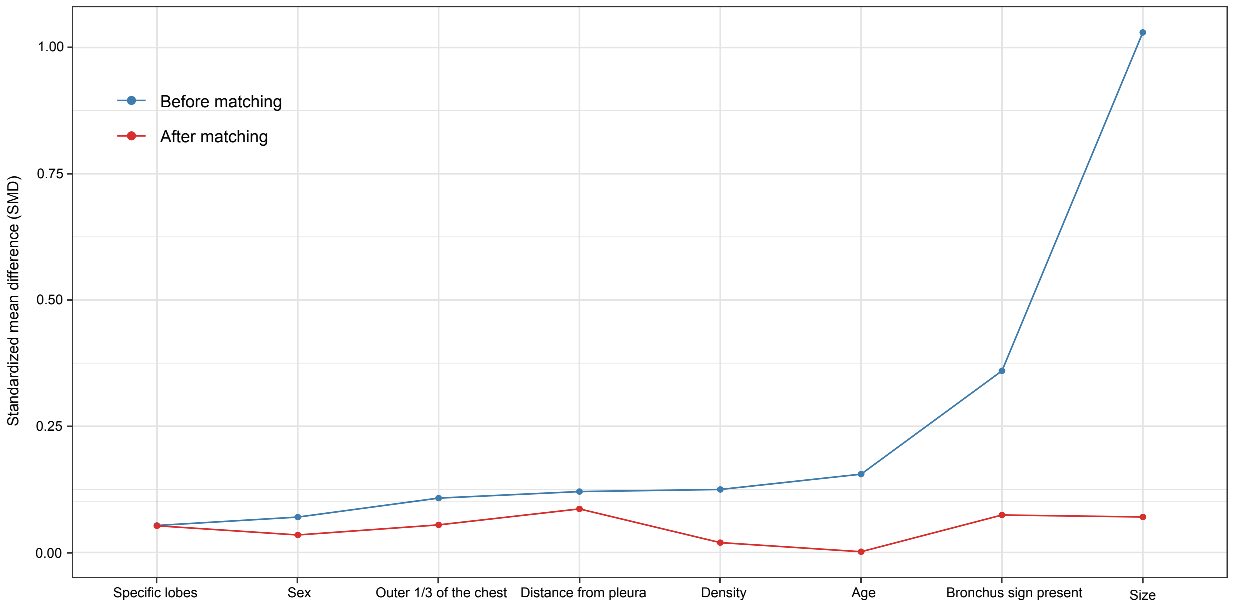

Figure E2. Covariate balance before and after propensity score matching.

Appendix E1. Pre-biopsy evaluation.

Before biopsy, all participants underwent thin-section chest CT to evaluate nodule characteristics. Neither positron emission tomography-computed tomography (PET-CT) nor multidisciplinary discussion (MDT) constituted a routine mandatory prerequisite before biopsy. PET-CT is not routinely reimbursed under the basic medical insurance system in China and was therefore used only as a supplementary examination when clinically indicated, such as when further assessment of malignancy risk or possible distant metastasis was required. Comprehensive preoperative cardiopulmonary assessment was also performed, including pulmonary function testing to evaluate respiratory reserve, as well as electrocardiography and echocardiography to assess cardiac functional reserve. The feasibility of biopsy and the choice of navigation modality were ultimately determined by the treating bronchoscopist on the basis of the overall pre-biopsy evaluation.
